# Supplementary material for: An open-source framework for large-scale, flexible evaluation of biomedical text mining systems
Source: J Biomed Discov Collab. 2008 Jan 29;3:1. doi: 10.1186/1747-5333-3-1 (PMC2276192; doi:10.1186/1747-5333-3-1)
Supplement: Additional file 1 — Evaluation platform source code. The additional file contains the software evaluation framework discussed in this paper. The most current release can be downloaded from SourceForge.net [1]. See the accompanying README file for instructions on installation and use. Also included in the distribution is a collection of UIMA wrappers for some commonly used BioNLP tools and annotated corpora. [file 1747-5333-3-1-S1.zip › bionlp_uima_repository-1.0.1/docs/api/edu/uchsc/ccp/uima/annotation/CCPAnnotationSet_Type.html]

CCPAnnotationSet\_Type (Center for Computational Pharmacology Util/UIMA API)


|  |  |  |
| --- | --- | --- |
| **Overview** \| **Package** \| **Class** \| **Tree** \| **Deprecated** \| **Index** \| **Help** | |  |
| **PREV CLASS**   **NEXT CLASS** | **FRAMES**    **NO FRAMES**     **All Classes** |  |
| SUMMARY: NESTED \| FIELD \| CONSTR \| METHOD | DETAIL: FIELD \| CONSTR \| METHOD |  |


---


## edu.uchsc.ccp.uima.annotation Class CCPAnnotationSet\_Type

```
java.lang.Object
  org.apache.uima.jcas.cas.TOP_Type
      edu.uchsc.ccp.uima.annotation.CCPAnnotationSet_Type
```

---

``` public class CCPAnnotationSet_Type extends org.apache.uima.jcas.cas.TOP_Type ```

The annotation set provides a means for arbitrarily categorizing or clustering groups of annotations. Annotations can be associated with multiple annotation groups. Examples of use include, defining Gold Standard annotation sets, and delineating between the use of different parameters during annotation, among others. Each annotation set is associated with a unique ID, a name and a description.
Updated by JCasGen Tue Aug 28 16:44:10 MDT 2007

---

| **Field Summary** | |
| --- | --- |
| `static boolean` | `featOkTst` |
| `static int` | `typeIndexID` |

**Fields inherited from class org.apache.uima.jcas.cas.TOP\_Type**

`casImpl, casType, casTypeCode, instanceOf_Type, jcas, ll_cas, lowLevelArrayBoundChecks, lowLevelTypeChecks, useExistingInstance`


**Constructor Summary**

`CCPAnnotationSet_Type(org.apache.uima.jcas.JCas jcas,
org.apache.uima.cas.Type casType)`
  
          initialize variables to correspond with Cas Type and Features


| **Method Summary** | |
| --- | --- |
| `String` | `getAnnotationSetDescription(int addr)` |
| `int` | `getAnnotationSetID(int addr)` |
| `String` | `getAnnotationSetName(int addr)` |
| `protected  org.apache.uima.cas.impl.FSGenerator` | `getFSGenerator()` |
| `void` | `setAnnotationSetDescription(int addr, String v)` |
| `void` | `setAnnotationSetID(int addr, int v)` |
| `void` | `setAnnotationSetName(int addr, String v)` |

**Methods inherited from class org.apache.uima.jcas.cas.TOP\_Type**

`addToIndexes, checkType, getTypeIndexID, invalidTypeArg, noObjCreate, removeFromIndexes`

**Methods inherited from class java.lang.Object**

`clone, equals, finalize, getClass, hashCode, notify, notifyAll, toString, wait, wait, wait`

**Field Detail**

### typeIndexID

```
public static final int typeIndexID
```

---


### featOkTst

```
public static final boolean featOkTst
```


**Constructor Detail**

### CCPAnnotationSet\_Type

```
public CCPAnnotationSet_Type(org.apache.uima.jcas.JCas jcas,
                             org.apache.uima.cas.Type casType)
```

:   initialize variables to correspond with Cas Type and Features


**Method Detail**

### getFSGenerator

```
protected org.apache.uima.cas.impl.FSGenerator getFSGenerator()
```

:   **Overrides:**: `getFSGenerator` in class `org.apache.uima.jcas.cas.TOP_Type`

---


### getAnnotationSetID

```
public int getAnnotationSetID(int addr)
```

---


### setAnnotationSetID

```
public void setAnnotationSetID(int addr,
                               int v)
```

---


### getAnnotationSetName

```
public String getAnnotationSetName(int addr)
```

---


### setAnnotationSetName

```
public void setAnnotationSetName(int addr,
                                 String v)
```

---


### getAnnotationSetDescription

```
public String getAnnotationSetDescription(int addr)
```

---


### setAnnotationSetDescription

```
public void setAnnotationSetDescription(int addr,
                                        String v)
```


---


|  |  |  |
| --- | --- | --- |
| **Overview** \| **Package** \| **Class** \| **Tree** \| **Deprecated** \| **Index** \| **Help** | |  |
| **PREV CLASS**   **NEXT CLASS** | **FRAMES**    **NO FRAMES**     **All Classes** |  |
| SUMMARY: NESTED \| FIELD \| CONSTR \| METHOD | DETAIL: FIELD \| CONSTR \| METHOD |  |


---
